# Prevalence of Anemia and Its Associated Factors Among 6–59‐Month‐Old Children in Ayder Comprehensive Specialized Referral Hospital, Northern Ethiopia

**DOI:** 10.1155/jnme/5114961

**Published:** 2026-08-06

**Authors:** Tigist Haile Gebrehiwot, Abadi Hailay Atsbaha, Shewit Engdashet Berhe

**Affiliations:** ^1^ Department of Public Health, College of Medicine and Health Science, Adigrat University, Adigrat, Ethiopia, adu.edu.et; ^2^ Department of Public Health Nutrition, College of Medicine and Health Science, Aksum University, Aksum, Ethiopia, aku.edu.et

**Keywords:** 6–59-month-old children, associated factor, anemia

## Abstract

**Introduction:**

Anemia is one of the most widespread diseases, with one in four people affected by anemia worldwide. In sub‐Saharan Africa, anemia in preschool children is considered a severe public health problem. This study aimed to assess the prevalence of anemia and its associated factors among 6–59‐month‐old children in Ayder Comprehensive Specialized Referral Hospital, Tigray, Northern Ethiopia.

**Methods:**

A hospital‐based cross‐sectional study was conducted among systematically selected 423 6–59‐month‐old children who were seeking service at Ayder Comprehensive Specialized Referral Hospital. Data were collected using a structured questionnaire. Four mL of venous blood from the finger was collected and analyzed using a Sysmex machine (model XP‐300). Anemia in children was defined as a hemoglobin level below 11 g/dL. Data were entered and analyzed using SPSS Version 21. Variables with a *p* value of < 0.2 in the bivariable analysis were selected for multivariable logistic regression, and statistical significance was declared at *p* value < 0.05 and CI of 95%.

**Results:**

The prevalence of anemia among children aged 6–59 months was 57.1% (95% confidence interval: 52.2%–62.1%); of this magnitude, mild, moderate, and severe anemia was 27.1%, 20.9%, and 9.1%, respectively. Morphological, microcytic hypochromic anemia was the most common type of anemia. Children aged 6–23 months (adjusted odds ratio = 2.374; 95% confidence interval: 1.428–3.945), stunting (adjusted odds ratio = 2.543; 95% confidence interval: 1.503–4.302), underweight (adjusted odds ratio = 6.660; 95% confidence interval: 3.046–14.566), and intestinal parasite infection (adjusted odds ratio = 2.803; 95% confidence interval: 1.242–6.322) were factors associated with anemia.

**Conclusion:**

The prevalence of anemia shows that it is a severe public health problem. Our findings underscore the need for nutrition counseling, deworming, growth monitoring, and promotion to minimize stunting, underweight, and intestinal parasite infection among children.

## 1. Introduction

Anemia is a condition that is characterized by a decreased level of hemoglobin (Hb), hematocrit, or red blood cells below the normal range for age, sex, physiological state, and altitude [1]. Anemia in preschool children is diagnosed as a hemoglobin level < 11 g/dL. According to the World Health Organization (WHO), public health significance of anemia is categorized as a no, mild, moderate, and severe public health problem when the prevalence of anemia in the community is < 5%, 5%–19.9%, 20%–39.9%, and ≥ 40%, respectively [2].

Anemia is one of the most widespread diseases, with one in four people affected by anemia worldwide. According to a WHO report, worldwide more than 1.62 billion people are anemic, and preschool children account for about 47% of the total anemic patients. Anemia is most prevalent in most parts of Africa, South Asia, and some countries in East Asia and the Pacific [3]. The highest magnitude of anemia is found in developing countries [4].

In sub‐Saharan Africa, anemia in preschool children is considered a severe public health problem [5]. According to Ethiopian Demography Health Survey (EDHS) 2016, in Ethiopia, the prevalence of anemia among children of 6–59 months of age increased from 44% in 2011 to 57% in 2016 [6]. In children, anemia significantly affects physical, mental, and social development and also changes their cognitive function. This in turn causes poor school performance, which in turn decreases their work productivity [7].

Anemia is caused by multiple factors, including micronutrient deficiencies like those of iron, folate, and vitamin B6 and B12 to infectious diseases such as malaria, different parasitic infections, and other noninfectious diseases [8]. Records from Ayder Comprehensive Specialized Referral Hospital show that most children under five suffer from infections, including hospital‐acquired ones. Infections play a significant role in interfering with nutrient intake and absorption, which results in anemia [3]. According to different studies, some of the factors for the high prevalence of anemia in Ethiopia were age, residency, maternal educational status, family income, early initiation of complementary food, underweight [9], stunting [10], and parasite infection [11].

Although the prevalence of anemia among hospitalized 6–59‐month‐old children has been studied in different parts of the world such as in India 63.3% [12], Brazil 56.6% [13], Southern Tanzania 83.17% [14], and Ghana 55% [15] and in different regions of Ethiopia such as in Gondar 54.1% [11] and South Wollo 41.1% [9], there is limited evidence in the Tigray region. Therefore, this study aimed to assess the prevalence of anemia and its associated factors among 6–59‐month‐old children in Ayder Comprehensive Specialized Referral Hospital, Tigray, Northern Ethiopia.

## 2. Materials and Methods

### 2.1. Study Design and Setting

A hospital‐based cross‐sectional study was conducted among 6–59‐month‐old children who were seeking service at Ayder Comprehensive Specialized Referral Hospital. Ayder Comprehensive Specialized Referral Hospital is located in Mekelle city, Tigray region, Northern Ethiopia. This hospital is the largest and the first specialized referral hospital in Tigray; it was launched in 2008, rendering its referral and nonreferral services to more than 8 million populations in its catchment areas of Tigray, Afar, and the north‐eastern parts of Amhara regional state. Mekelle city is located at a distance of 780 km from the capital city of Ethiopia, Addis Ababa, and it is found at 2254 m above sea level. Every year, more than 13,500 children 6–59 months of age visit this hospital.

### 2.2. Study Population

All 6‐ to 59‐month‐old children with their parents/caregivers came to the pediatric outpatient, emergency, or inpatient at Ayder Comprehensive Specialized Referral Hospital during the data collection period. Children 6–59 months of age with active bleeding, a history of blood transfusion, and/or children who have undergone surgery were excluded from the study.

### 2.3. Sample Size Determination

The sample size was calculated using a single population proportion formula, assuming a 50% proportion of anemia among 6–59‐month‐old children (since no study is conducted at the hospital level in the area or the region), a 5% margin of error, and a 95% confidence interval (CI). After adding a 10% nonresponse rate, the final sample size was 423.

### 2.4. Sampling Procedures

The under‐five outpatient department, emergency, and inpatient departments of Ayder Comprehensive Specialized Referral Hospital get an average visitor of 1150 children in one month. The total sample size was taken from the three wards based on proportion to the population size of the number of children who came to each department per month. The *k*th interval was determined (*k* = the number of children visiting under five outpatient, emergency, and inpatient departments within one month before the study (1150)/sample size (423 = 2.72 = 3)). Then, using a systematic random sampling technique, every third child was taken. The first child from each department was selected randomly between 1 and 3 children by the lottery method.

### 2.5. Data Collection Procedures

Data were collected through a face‐to‐face interview using a structured questionnaire, which was adapted and developed from EDHS 2016 [6] and other relevant research [9, 11]. The questionnaire was designed to acquire information concerning sociodemographic, nutritional, and disease‐related factors.

The questionnaire was prepared in English and translated into the local language (Tigrigna), and then, it was returned to English to ensure consistency of concepts. A pretest was conducted in 5% of the sample size in Mekelle hospital.

Anthropometric measurement of the child’s length, height, and weight was conducted. For children aged 6–23 months, recumbent length was taken, and for 24–59 months, standing height was measured recording to the nearest 0.1 cm. The weight of the children was measured using an electronic body scale (model Tcs‐150‐RT) to the nearest 0.1 kg. Data collectors first adjusted the scale to zero and removed the child’s hot shoes and heavy outer clothing before weighing every child. Measurements were done twice for every child, and an average was taken to ensure accuracy. Analysis of anthropometric data was done using Emergency Nutrition Assessment (ENA) Version: Oct/07 software 2006 WHO standards. Based on anthropometric measurement, children were considered underweight if their weight‐for‐age *z*‐score was below −2 standard deviation (SD), stunted if their length/height‐for‐age *z*‐score was below −2 SD, and wasted if their weight‐for‐length/height *z*‐score was below −2 SD, according to the WHO child growth standards [16].

Four mL of venous blood from the finger was collected through an ethylene diamine tetraacetic acid test tube from each child to calculate the complete blood count (CBC). Blood was analyzed using a Sysmex machine (model XP‐300), and the machine washing was done every day with a cleaning solution. As recommended by WHO, hemoglobin results were adjusted by subtracting 0.8 g/dL from its respective sea level because the study area is found at 2254 m above sea level. Then, anemia in children was defined as a hemoglobin level below 11 g/dL, and it was classified into mild (10‐< 11 g/dL), moderate (7–< 10 g/dL), and severe (below 7 g/dL) anemia [2].

Morphologically, anemia was further categorized into different types using corpuscular or cell volume (MCV) and mean cell hemoglobin (MCH). For 6–23‐month‐old children, MCV was classified as normal when it was between 72–88 fL and 76–90 fL for 24–59 months. Also, the MCH result was considered normal when it was 24–30 pg/cell, 25–31 pg/cell for up to 23 months, and 24–59 months, respectively [17].

In this study, a light microscope with thick blood film was used to diagnose the presence or absence of malaria parasites. After the thick blood formed, it was allowed to dry, and then, the slides were fixed with methanol reagent and stained with Giemsa stain, allowed to dry, washed with distilled water, and finally observed under a light microscope using oil immersion objectives (× 100). The presence or absence of malaria was reported when any parasites were detected in the blood smear.

A stool sample collection kit was also given to the child’s primary caregiver, and 1 g for the formed or 0.5–1 mL if it was a liquid (diarrhea) form of fresh stool specimen, was taken and observed under a microscope using normal saline objectives (× 10 or × 40) to see the presence or absence of intestinal parasitic infections in the light microscope.

### 2.6. Data Analysis

Data was coded, entered, cleaned, edited, and analyzed using Statistical Package for Social Science (SPSS) Version 21. Frequency and percentage were used to summarize categorical variables, while continuous and non‐normally distributed variables were summarized by median with interquartile range. Variables with a *p* value of < 0.2 in the bivariable analysis were selected for multivariable logistic regression, and statistical significance was declared at *p* value < 0.05 and CI of 95%.

### 2.7. Ethical Consideration

The study was approved by the institutional review board of Mekelle University, College of Health Sciences, School of Public Health Research Ethics Committee (approval no: 1319//2018). In addition, a support letter was obtained from the medical director of Ayder Comprehensive Specialized Referral Hospital. Written informed consent was taken from children’s parents/caregivers after the objective of the study was explained to them. Confidentiality of the study participants was kept. The right of the participant not to participate or to stop at any time they wanted was also kept. To decrease risk during blood sample collection, laboratory technologists followed the standard operating procedure.

## 3. Results

### 3.1. Sociodemographic Characteristics of Participants

A total of 406 children participated in the study with a 96% response rate. The median age of children was 24 months, with an interquartile range of 28.25 months. One hundred forty‐seven (36.2%) children were within the age range of 6–23 months. Two hundred five (50.5%) of the children were females, and 71.4% of the children came from urban areas.

Three hundred sixty‐four (89.7%) of the respondents were mothers. The median age of mothers or caregivers was 29 years, which ranged from 17 to 49 years. Three hundred forty‐one (84.0%) of the mothers or caregivers were married, and 88.7% of the mothers or caregivers were Orthodox Christians. The median household family size was 3, ranging from 2 to 9 (Table 1).

**TABLE 1:** Sociodemographic characteristics of the family and children aged 6–59 months in Ayder Comprehensive Specialized Referral Hospital, Northern Ethiopia.

Variables	Category	Frequency (*n*)	Percentage (%)
Age of child	6–23 months	147	36.2
	24–59 months	259	63.8

Sex of child	Male	201	49.5
	Female	205	50.5

Residency	Rural	116	28.6
	Urban	290	71.4

Respondent’s relation to the child	Mother	364	89.7
	Other^∗^	42	10.3

Marital status of mother/caregiver	Married	341	84.0
	Widowed	14	3.4
	Separated	23	5.7
	Divorced	28	6.9

Ethnicity of mother/caregiver	Tigre	370	91.1
	Amhara	26	6.4
	Afar	10	2.5

Religion of mother/caregiver	Orthodox Christians	360	88.7
	Muslim	43	10.6
	Protestant	3	0.7

Educational status of mother/caregiver	No formal education	97	23.9
	Primary education	116	28.6
	≥ Secondary education	193	47.5

Occupational status of mother/caregiver	Housewife	213	52.5
	Other	193	47.5

Family size	≤ 3	210	51.7
	4‐6	170	41.9
	> 6	26	6.4

^∗^Father, grandparents.

### 3.2. Nutrition‐Related Characteristics of Children Aged 6–59 Months

Three hundred and seven (97.8%) of the children had a history of breastfeeding since their birth, and 219 (53.9%) of the children were introduced to complementary feeding at 6–8 months of age. Three hundred twenty‐one (79.1%) children were taking tea immediately after or with meals. One hundred sixty‐three (40.1%), 99 (24.4%), and 38 (9.4%) children were stunted, underweight, and wasted, respectively (Table 2).

**TABLE 2:** Nutrition‐related characteristics of children aged 6–59 months in Ayder Comprehensive Specialized Referral Hospital, Northern Ethiopia.

Variables	Category	Frequency (*n*)	Percentage (%)
Ever breastfeed	Yes	397	97.8
	No	9	2.2

Exclusively breastfed	Yes	237	58.4
	No	169	41.6

Introduction of complementary foods	Early (< 6 months)	81	20.0
	Timely (6–8 months)	219	53.9
	Late (≥ 9 months)	106	26.1

Tea intake with or right after a meal	Yes	321	79.1
	No	85	20.9

Length/height for age	Stunted	163	40.1
	Normal	243	59.9

Weight for age	Underweight	99	24.4
	Normal	307	75.6

Weight for length/height	Wasted	38	9.4
	Normal	368	90.6

### 3.3. Disease‐Related Characteristics of Children Aged 6–59 Months

Fifty‐eight (14.3%) and 56 (13.8%) children had histories of fever and diarrhea, respectively, two weeks before data collection. 17.5% of the children had intestinal parasites (Table 3).

**TABLE 3:** Disease‐related characteristics of children aged 6–59 months in Ayder Comprehensive Specialized Referral Hospital, Northern Ethiopia.

Variables	Category	Frequency (*n*)	Percentage (%)
History of fever in the past 2 weeks	Yes	58	14.3
	No	348	85.7

History of diarrhea in the past 2 weeks	Yes	56	13.8
	No	350	86.2

Current diarrhea	Yes	95	23.4
	No	311	76.6

History of malaria infection in a lifetime	Yes	11	2.7
	No	395	97.3

Current malaria parasitemia	Yes	3	0.7
	No	403	99.3

Current intestinal parasites	Yes	71	17.5
	No	335	82.5

### 3.4. The Prevalence of Anemia Among Children Aged 6–59 Months

Two hundred thirty‐two, 57.1% (95% CI: 52.2%–62.1%), children were anemic. The prevalence of mild, moderate, and severe anemia was 27.1%, 20.9%, and 9.1%, respectively. In male children, the prevalence of anemia was 62.2%, whereas among female children, it was 52.2%. The highest prevalence of anemia was found in the age group of 6–23 month children.

Of the overall 6–59 months old anemic children, 47.8% had microcytic hypochromic anemia, 37.9% had normocytic normochromic anemia, 5.6% had macrocytic normochromic anemia, 4.7% had macrocytic hypochromic anemia, 3.0% had macrocytic hyperchromic anemia, and 0.9% had microcytic normochromic anemia.

### 3.5. Factors Associated With Anemia

Variables with *p* value < 0.2 in bivariable analysis (child age, sex of child, residency, marital status of mother/caregiver, educational level of mother/caregivers, family size, exclusive breastfeeding, time of introduction of complementary foods, stunting, underweight, wasting, history of fever in the past 2 weeks, current diarrhea, and current intestinal parasites) were entered to multivariable logistic regression. During multivariable analysis being in the age range of 6–23 months, stunting, being underweight, and intestinal parasite infection were significantly associated with anemia (Table 4).

**TABLE 4:** Factors associated with anemia among children aged 6–59 months in Ayder Comprehensive Specialized Referral Hospital, Northern Ethiopia.

Variables	Anemia status		COR (95% CI)	AOR (95% CI)
	Yes (*n* = 232)	No (*n* = 174)		
*Age of children*				
6–23 months	101 (68.7%)	46 (31.3%)	2.145 (1.402–3.283)	2.374 (1.428–3.945)^∗∗^
24–59 months	131 (50.6%)	128 (49.4)	1	1

*Sex of child*				
Male	125 (62.2%)	76 (37.8%)	1.506 (1.014–2.237)	1.254 (0.769–2.044)
Female	107 (52.2%)	98 (47.8%)	1	1

*Residency*				
Rural	92 (79.3%)	24 (20.7%)	4.107 (2.479–6.804)	1.666 (0.797.‐3.482)
Urban	140 (48.3%)	150 (51.7%)	1	1

*Marital status of mother/caregiver*				
Married	186 (54.5%)	155 (45.5%)	0.400 (0.166–0.966)	0.638 (0.218–1.868)
Widowed	8 (57.1%)	6 (42.9%)	0.444 (0.114–1.733)	0.941 (0.196–4.524)
Separated	17 (73.9%)	6 (26.1%)	0.944 (0.267–3.343)	1.138 (0.236–5.489)
Divorced	21 (75.0%)	7 (25.0%)	1	1

*Educational status of the of the mother/caregiver*				
No formal education	77 (79.4%)	20 (20.6%)	4.406 (2.498–7.771)	1.484 (0.644–3.420)
Primary education	65 (56.0%)	51 (44.0%)	1.459 (0.918–2.318)	1.072 (0.603–1.905)
≥ Secondary education	90 (46.6%)	103 (53.4%)	1	1

*Family size*				
≤ 3	109 (51.9%)	101 (48.1%)	0.925 (0.409–2.094)	0.599 (0.222–1.616)
4–6	109 (64.1%)	61 (35.9%)	1.532 (0.666–3.521)	0.817 (0.298–2.238)
> 6	14 (53.8%)	12 (46.2%)	1	1

*Exclusive breastfeeding*				
Yes	113 (47.7%)	124 (52.3%)	0.383 (0.252–0.581)	0.771 (0.395–1.503)
No	119 (70.4%)	50 (29.6%)	1	1

*Introduction of complementary foods*				
Early (< 6 months)	60 (74.1%)	21 (25.9%)	3.400 (1.935–5.974)	1.715 (0.699–4.206)
Late (≥ 9 months)	72 (67.9%)	34 (32.1%)	2.520 (1.549–4.101)	1.300 (0.682–2.480)
Timely (6–8 months)	100 (45.7%)	119 (54.3%)	1	1

*Length/height for age*				
Stunted	127 (77.9%)	36 (22.1%)	4.637 (2.960–7.262)	2.543 (1.503–4.302)^∗∗^
Normal	105 (43.2%)	138 (56.8%)	1	1

*Weight for age*				
Underweight	89 (89.9%)	10 (10.1%)	10.207 (5.114–20.372)	6.660 (3.046–14.566)^∗∗^
Normal	143 (46.6%)	164 (53.4%)	1	1

*Weight for length/height*				
Wasted	31 (81.6%)	7 (18.4%)	3.679 (1.580–8.570)	1.706 (0.587–4.957)
Normal	201 (54.6%)	167 (45.4%)	1	1

*History of fever in the past 2 weeks*				
Yes	38 (65.5%)	20 (34.5%)	1.508 (0.843–2.697)	0.587 (0.263–1.309)
No	194 (55.7%)	154 (44.3%)	1	1

*Current diarrhea*				
Yes	63 (66.3%)	32 (33.7%)	1.654 (1.023–2.674)	1.357 (0.748–2.463)
No	169 (54.3%)	142 (45.7%)	1	1

*Current intestinal parasite infection*				
Yes	58 (81.7%)	13 (18.3%)	4.128 (2.180–7.817)	2.803 (1.242–6.322)^∗^
No	174 (51.9%)	161 (48.1%)	1	1

Abbreviations: AOR, adjusted odds ratio; COR, crude odds ratio.

^∗^(*p* value of 0.001–0.05).

^∗∗^(*p* value of ≤ 0.001); 1: reference group.

## 4. Discussion

This study assessed the prevalence and associated factors of anemia among 6–59‐month‐old children in the Ayder Comprehensive Specialized Referral Hospital, in Northern Ethiopia. According to this study, the prevalence of anemia was 57.1% (95% CI: 52.2%–62.1%); this prevalence is classified as a severe public health problem according to the WHO classification of the public health significance of anemia [2]. The result is consistent with the findings of studies conducted among hospitalized children in Gondar, Ethiopia [11], Uganda [18], Ghana [15], and Brazil [13], which reported anemia prevalence of 54.1%, 56.3%, 55%, and 56.6%, respectively. However, our finding is lower than the findings of studies conducted in Mwanza, Tanzania (77.2%) [19], India (77.8%) [20], Lebanon (71.9%) [21], and southern Tanzania (83.2%) [14]. This difference might be due to heterogeneity of the populations, sample size differences, and differences in the nutritional status of the children.

Children aged 6–23 months were 2.37 times ([AOR] = 2.374, 95% CI: 1.428–3.945) more likely to have anemia as compared to children aged 24–59 months old. This finding is supported by studies conducted in Bangladesh [22], Cape Verde, and West Africa [23]. This can be justified by increased iron need due to fast growth in the first 2 years and low availability of iron‐rich food [24], and as age increases, iron intake is also likely to increase because of the probability of increased diversity of foods [25].

The study showed that stunted children were 2.5 times (95% CI: 1.503–4.302) more likely to have anemia than normal‐stature children. This result is consistent with studies conducted in Ethiopia [10] and Kenya [26]. Underweight children had also higher odds ([AOR] = 6.660, 95% CI: 3.046–14.566) of developing anemia than children with normal weight. This result was in line with a study conducted in South Wollo [9], Kilte Awulaelo Woreda, Ethiopia [27], and Rwanda [28]. This might be because the combined effect of micronutrient deficiency, stunting [29], and underweight causes anemia.

Children with intestinal parasite infection were 2.8 times (95% CI: 1.242–6.322) more likely to be affected by anemia than children with no parasite infection. A similar result was observed in a study conducted in Gondar, Ethiopia [11], and West Malaysia [30]. This may be because intestinal parasites can cause blood loss and compete for nutrients ultimately causing anemia [31].

## 5. Conclusions

The prevalence of anemia among hospitalized 6–59‐month‐old children was found to be 57.1%, which shows severe public health significance. Children aged 6–23 months, stunting, being underweight, and intestinal parasite infection were independent determinants of anemia. In conclusion, our findings underscore the need for nutrition counseling, deworming, growth monitoring, and promotion to minimize stunting, underweight, and intestinal parasite infection among children.

## Author Contributions

Conceptualization, investigation, data curation, writing–review and editing, and project administration: Tigist Haile Gebrehiwot, Abadi Hailay Atsbaha, and Shewit Engdashet Berhe; methodology and formal analysis, Tigist Haile Gebrehiwot and Shewit Engdashet Berhe; resources, Abadi Hailay Atsbaha; writing–original draft preparation, Tigist Haile Gebrehiwot; supervision, Abadi Hailay Atsbaha and Shewit Engdashet Berhe. All authors contributed writing, reading, and editing of the manuscript.

## Funding

No funding was obtained for this study.

## Conflicts of Interest

The authors declare no conflicts of interest.

## Data Availability

The data used to support the findings of this study will be made available by authors upon reasonable request.
